# An agent-based model of tsetse fly response to seasonal climatic drivers: Assessing the impact on sleeping sickness transmission rates

**DOI:** 10.1371/journal.pntd.0006188

**Published:** 2018-02-09

**Authors:** Simon Alderton, Ewan T. Macleod, Neil E. Anderson, Gwen Palmer, Noreen Machila, Martin Simuunza, Susan C. Welburn, Peter M. Atkinson

**Affiliations:** 1 Geography and Environment, Faculty of Social and Human Sciences, University of Southampton, Southampton, United Kingdom; 2 Lancaster Environment Centre, Lancaster University, Lancaster, United Kingdom; 3 Centre for Health Informatics, Computing and Statistics (CHICAS), Lancaster Medical School, Lancaster University, Lancaster, United Kingdom; 4 Division of Infection and Pathway Medicine, Biomedical Sciences, Edinburgh Medical School, College of Medicine and Veterinary Medicine, The University of Edinburgh, Edinburgh, United Kingdom; 5 The Royal (Dick) School of Veterinary Studies and the Roslin Institute, University of Edinburgh, Roslin, United Kingdom; 6 Independent Researcher, Leyland, Lancashire, United Kingdom; 7 Department of Disease Control, School of Veterinary Medicine, University of Zambia, Lusaka, Zambia; 8 School of Geography, Archaeology and Palaeoecology, Queen's University Belfast, Northern Ireland, United Kingdom; 9 State Key Laboratory of Resources and Environmental Information System, Institute of Geographic Sciences and Natural Resources Research, Chinese Academy of Sciences, Beijing, China; Institut de recherche pour le developpement, FRANCE

## Abstract

**Background:**

This paper presents the development of an agent-based model (ABM) to incorporate climatic drivers which affect tsetse fly (*G*. *m*. *morsitans*) population dynamics, and ultimately disease transmission. The model was used to gain a greater understanding of how tsetse populations fluctuate seasonally, and investigate any response observed in *Trypanosoma brucei rhodesiense* human African trypanosomiasis (rHAT) disease transmission, with a view to gaining a greater understanding of disease dynamics. Such an understanding is essential for the development of appropriate, well-targeted mitigation strategies in the future.

**Methods:**

The ABM was developed to model rHAT incidence at a fine spatial scale along a 75 km transect in the Luangwa Valley, Zambia. The model incorporates climatic factors that affect pupal mortality, pupal development, birth rate, and death rate. In combination with fine scale demographic data such as ethnicity, age and gender for the human population in the region, as well as an animal census and a sample of daily routines, we create a detailed, plausible simulation model to explore tsetse population and disease transmission dynamics.

**Results:**

The seasonally-driven model suggests that the number of infections reported annually in the simulation is likely to be a reasonable representation of reality, taking into account the high levels of under-detection observed. Similar infection rates were observed in human (0.355 per 1000 person-years (SE = 0.013)), and cattle (0.281 per 1000 cattle-years (SE = 0.025)) populations, likely due to the sparsity of cattle close to the tsetse interface. The model suggests that immigrant tribes and school children are at greatest risk of infection, a result that derives from the bottom-up nature of the ABM and conditioning on multiple constraints. This result could not be inferred using alternative population-level modelling approaches.

**Conclusions:**

In producing a model which models the tsetse population at a very fine resolution, we were able to analyse and evaluate specific elements of the output, such as pupal development and the progression of the teneral population, allowing the development of our understanding of the tsetse population as a whole. This is an important step in the production of a more accurate transmission model for rHAT which can, in turn, help us to gain a greater understanding of the transmission system as a whole.

## Author summary

African trypanosomiasis is a parasitic disease which affects humans and other animals in 36 sub-Saharan African countries. The disease is transmitted by the tsetse fly, and the human form of the diseases is known as sleeping sickness. In an attempt to improve our understanding of the mechanisms which contribute to sleeping sickness transmission, a detailed, seasonally driven model of the tsetse fly has been produced, with the theory that a greater understanding of the disease vector’s life cycle will allow developments in our knowledge of disease transmission. The model incorporates previously developed spatial data for the Luangwa Valley case study, along with demographic data for its inhabitants. Tsetse and potential human and animal hosts are modelled at the individual level, allowing each contact and infection to be recorded through time. Through modelling at a fine-scale, we can incorporate detailed mechanisms for tsetse birth, feeding, reproduction and death, while considering what demographics, and which locations, have a heightened risk of disease.

## Introduction

The tsetse fly (genus: *Glossina*) is the vector for human African trypanosomiasis (HAT) or sleeping sickness, a neglected tropical disease caused by two sub-species of the protozoan parasite *Trypanosoma brucei* s.l.: *T*. *b*. *rhodesiense*, in eastern and southern Africa and *T*. *b*. *gambiense* in West Africa [1]. *T*. *b*. *rhodesiense* HAT (rHAT) is a zoonosis, affecting a wide range of wildlife [2,3] and domestic animals, particularly cattle [4], presenting in humans as an acute disease [5]. The history of HAT in sub-Saharan Africa is characterised by long periods of endemicity where the disease self-sustains at low background levels, with periodic epidemics in regional *foci* [6]. As sleeping sickness is a neglected tropical disease, treatments are often out-of-date, difficult to administer, physically invasive and partially validated, with the prospect for future developments of more effective treatments being limited (e.g. [7–11]). Furthermore, where tools are available, HAT is rarely prioritised due to competing public health interests [12]. In terms of disease prevention, there is currently no immunological prophylaxis to stop infection in humans [13], made difficult to produce due to the parasite being able to evade the host's immune response by altering the antigenic character of its glycoprotein surface coat [14]. Given these difficulties with preventing and treating HAT infection in humans, it is not surprising that mitigation strategies focused on vector control have seen success (e.g. [15–18]), given that the tsetse fly is not only required for transmission, but also for several stages of parasite development [19,20]. Despite such efficacy, the control of the disease in tsetse (and, therefore, wildlife) in game reserves and other protected areas is complicated by ecological, conservationist and environmental considerations [21–23].

Gaining a greater understanding of the population dynamics in a tsetse population appears to be an attractive goal, considering that such an understanding could lead to the development of more targeted vector control strategies which have a less adverse ecological impact, while also allowing a more plausible understanding of the rHAT transmission system. For the latter, demographic growth (through the availability of food and habitat) and climate changes (affecting tsetse development and mortality rates) are two factors which could affect tsetse population dynamics, and ultimately affect the transmission system [24,25]. As a result of the significant role that a tsetse population has in determining the rate and distribution of rHAT transmission, this paper considers the tsetse sub-component of the larger rHAT transmission system in detail, with the ultimate goal being the creation of a more accurate representation of the transmission system as a whole.

Collecting comprehensive data on populations of tsetse in the field is expensive, complex and time consuming and, consequently, numerous attempts have been made to model tsetse populations as part of vector control or HAT transmission studies (e.g. [26–29]). Some models incorporate climatic drivers which create fluctuations in the tsetse population through the seasons (e.g. [30–33]). One recent example used agent-based modelling (ABM) techniques to simulate a simple fluctuation in tsetse population size through different seasons by altering the length of a predetermined lifespan for tsetse, depending on whether the tsetse emerges in the dry (2 months) or wet season (3 months) [31]. Incorporating more detail, [33] used known relationships between temperature and different life events and processes, such as mortality and the length of the pupation period, as parameters when constructing a population model for vector control.

ABMs are “a computerized simulation of a number of decision-makers or agents, and institutions, which interact through prescribed rules” [34]. ABMs have been described as a “third way” of conducting scientific research, incorporating both deductive since ABMs start with basic assumptions, and inductive approaches, as they produce simulation data to analyse [35]. However, Epstein [36] suggests that rather than inductive or deductive, ABMs should be considered as “generative” tools in that, through the initialisation of a population of autonomous agents in a relevant spatial environment, one can allow the agents to interact given a simple set of local rules, and generate, from the bottom up, the macroscopic behaviour and regularity of the population as a whole. Such an approach lends itself well to both the investigation of the HAT transmission system as a whole and the tsetse populations and their dynamics as a component. Starting with tsetse population dynamics, much is written about how varying climatic conditions have different impacts on various tsetse life events and processes e.g.: pupal period duration (e.g. [37]), probability of pupal death (e.g. [38,39]), and time between oviposition (e.g. [40,41]). Representing observations made from samples acquired both in the field and laboratory studies, these patterns provide us with a solid framework to model the larger population, for which comprehensive data are much more difficult, if not impossible, to acquire. By initialising a tsetse population as individuals, each abiding by rules set by the above behavioural patterns (and others relating to feeding, mating and age-dependent mortality), plausible population level outcomes such as fluctuations in population size should be observable as the simulation progresses.

When the HAT transmission system is incorporated into an ABM for acquiring preliminary knowledge of the disease transmission system, the constructed model becomes a representation of a complex system (e.g. [42–44]), given that the prevalence of the disease is a complicated emergent phenomenon produced by relatively simple, individual specific rules (both vector and host) concerning movement and resource acquisition. In a complex system, the causes of emergent phenomena cannot easily be decoupled and explained by specific parts of the system [45] with, in this case, the model landscape and agent behaviour creating variation in the timing, location and probability of infection as a result of their influence on variability in contact patterns between vector and host [46,47]. In this way, ABMs could be considered the most appropriate way to investigate both the HAT transmission system, and tsetse fly dynamics as a sub-component, allowing the representation of interdependent processes such as how individuals interact with each other and their environment through space and time more easily than is possible through more traditional epidemiological techniques [48].

In previous work, an ABM of rHAT transmission was produced using a spatialized approach, incorporating factors often overlooked (e.g. human behaviour and activity-based movement; density and mobility of vectors; and the contribution of additional hosts) [27]. This paper presents the first ABM which considers the effect of climatic factors on individual tsetse and their life processes in detail, while also considering the effect this has on rHAT transmission in a large study area in Eastern Province, Zambia. Through the incorporation of seasonality parameters into an existing fine spatial and temporal scale ABM of rHAT transmission in the region [27], the aim was to develop a greater understanding of tsetse population dynamics through simulation, and subsequently produce a more plausible model of rHAT transmission. The incorporation of such data is vital where transmission rates, and indeed the transmission system as a whole, are to be explored over multiple years. The existing model provided a suitable starting point for the simulation of these seasonal parameters by modelling tsetse flies at the individual level, along with different life events for which durations and probability of occurrence can be climatically constrained. Ultimately, the modified model was implemented with the aim of answering the following research questions: throughout the year, how does the tsetse fly population fluctuate both as a whole, and within different life stages (e.g. pupal, teneral, mature)? Under the caveat that a plausible model has been produced, what rates of disease transmission are observed, and how do these vary seasonally? Such a model will allow for future exploration of long-term mitigations strategies, alterations to the demographic make-up of the study area, and climate change scenarios.

## Methods

### Study area

Eastern Province, Zambia is situated in southern Africa, sharing borders with Malawi (to the East) and Mozambique (to the South). The Luangwa Valley is an extension of the Great Rift Valley of East Africa, traversing the Zambian Eastern, Northern and Muchinga Provinces. The valley is characterised as a flat bottomed valley bounded by steep, dissected escarpments which rise to a plateau at approximately 900–1000 m [49]. Different types of vegetation are observed at different altitudes, with valley areas consisting mainly of mopane woodland and patches of grassland, while the natural vegetation on the escarpment and plateau is miombo woodland, interspersed with munga woodland [50].

**Fig 1:**
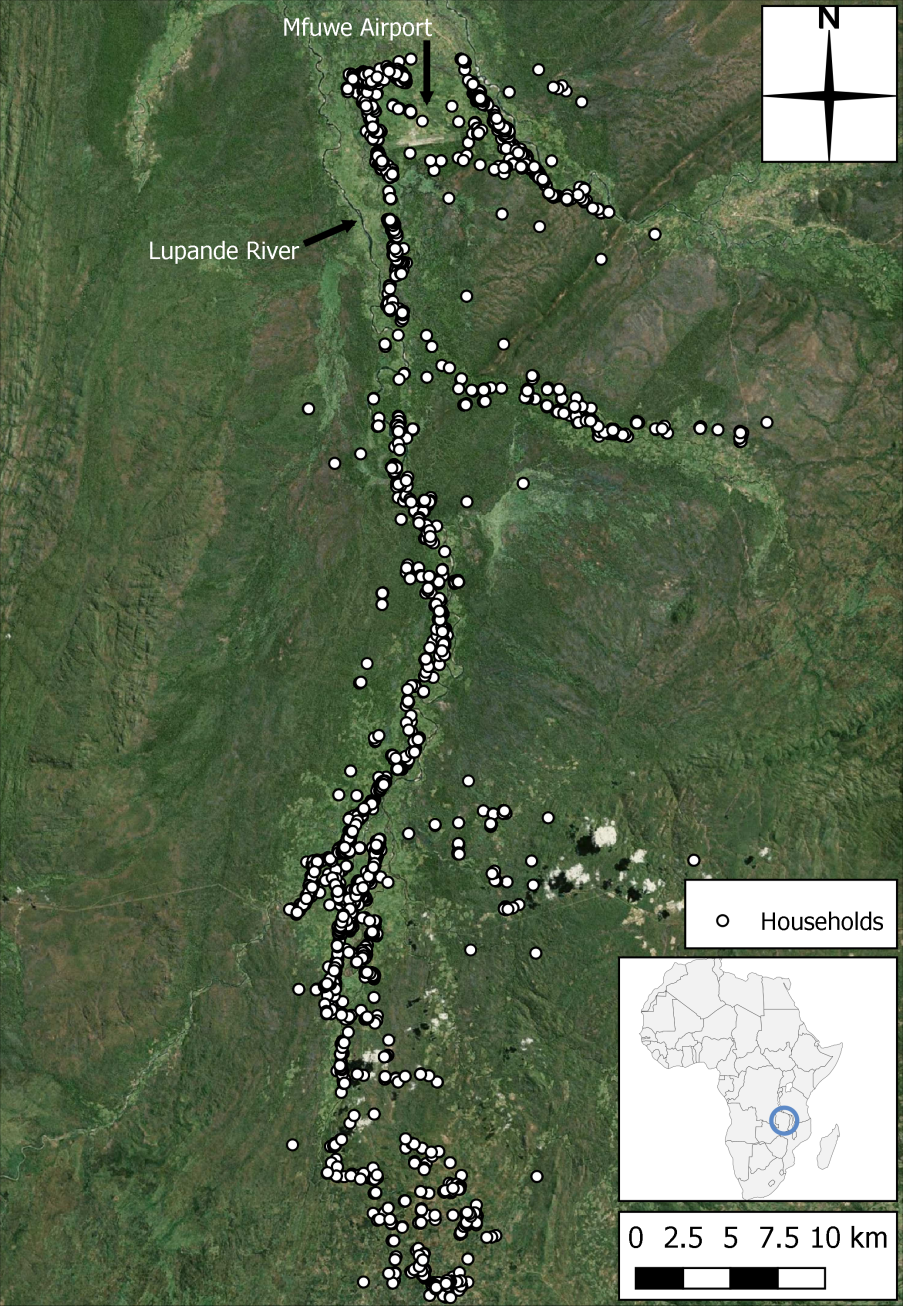
Map of the study area size and location. **Households from the census included in this modelling study are indicated as white circles and Mfuwe airport, the location of the region's weather station, is visible in the north (produced using Landsat 7 imagery from USGS), after** [27].

**Fig 2:**
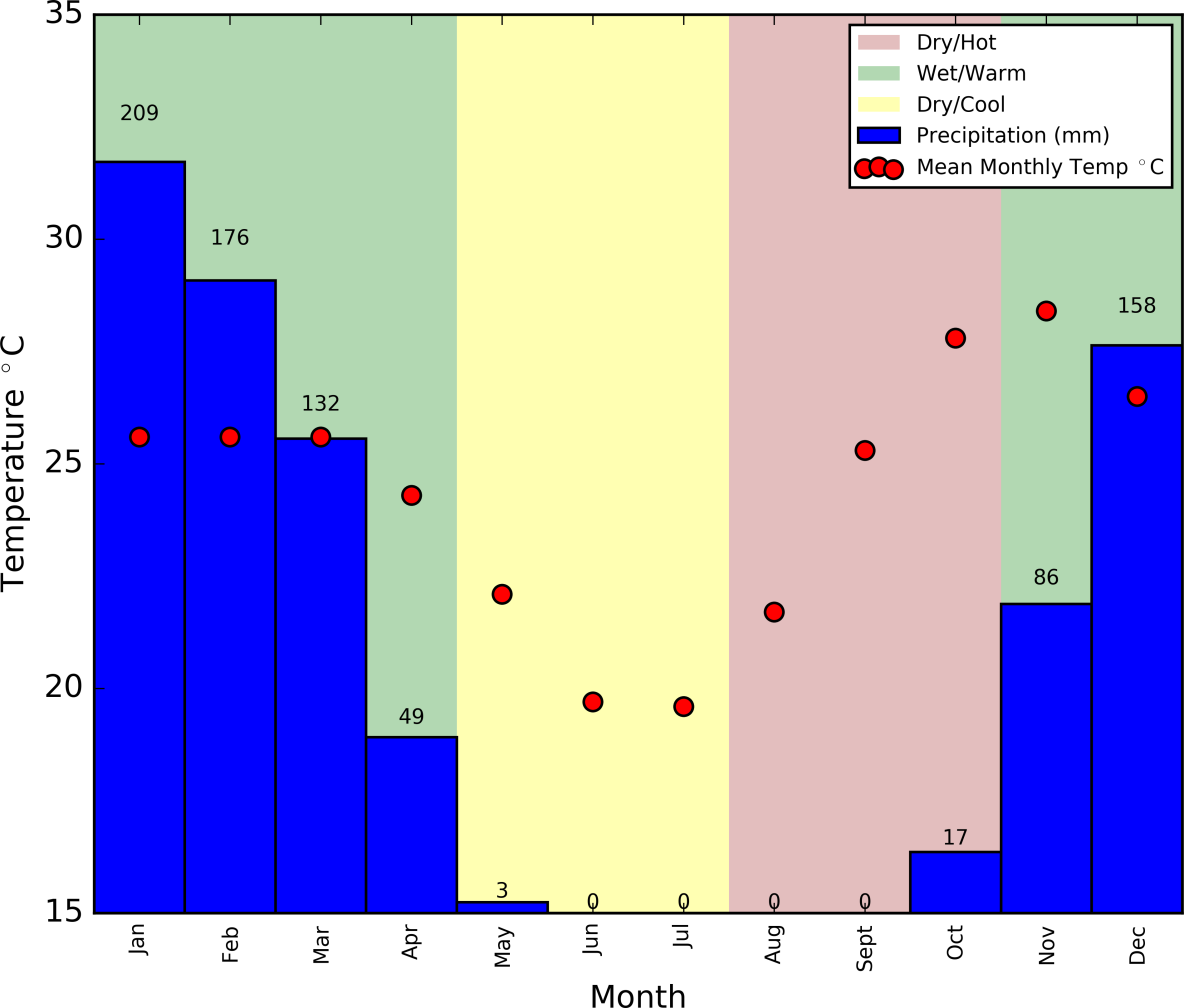
Average annual climate data for the study area, collected at Mfuwe airport weather station. **Produced using data from** [51]**. Background colours represent typical seasons found in the study area**.

The study area spans a sparsely populated region of the Luangwa Valley. Villages are small (between 5 and 20 households) and inhabitants are predominantly subsistence farmers. The data collection area and region to be modelled consists of a 75 km transect which starts close to Mfuwe airport in the north, and runs southwards along the Lupande River and its distributaries (Fig 1). Average monthly temperature and rainfall measurements collected at the Mfuwe airport (1982–2012) weather station are reproduced in Fig 2 [51]. There are three main seasons in Zambia’s tropical climate: the rainy season spans November to April (wet and warm) with mean monthly rainfall peaking at 210 mm in January. After the rains, a cold and dry period occurs prior to August, in which May is the hottest and wettest month, with mean temperatures below 23°C and mean rainfall below 3 mm. The hot and dry season usually spans August, September and October, with mean temperatures reaching 28°C in October accompanied by 17 mm of rainfall on average, the first after four dry months in succession [49,51]. The Luangwa River and its main tributaries are perennial, and although flash flooding occurs in all rivers during the wet season, the smaller rivers which drain the valley floor dry out during the dry season and flow during the rains [52].

rHAT is endemic in the Luangwa Valley, first being reported in 1908 [53]. *G*. *m*. *morsitans* was not originally considered a vector of rHAT in the valley, despite 50% of domestic and game animals in the Valley having been observed to harbour trypanosomes [54]. In the early 1970s, a large rHAT outbreak occurred in Isoka (241 case in 3 years) attributed to fly encroachment from Luangwa [55]. Wildlife had been observed to reside in Isoka for several months during the rainy season, migrating away during the dry season. In 1973, early diagnosis and improved treatment methods were introduced, and case numbers fell [56].

Today, cases of rHAT continue to be reported in the Luangwa Valley. Mid-Luangwa Valley has recently experienced increased immigration of people seeking fertile land. Land pressure has resulted in human settlement in increasingly marginal, tsetse-infested areas, previously avoided for fear of disease risk to introduced livestock. Households grow cotton as a cash crop and maize and groundnuts for home consumption [49]. These anthropogenic changes have the potential to destabilise current trypanosomiasis transmission cycles, resulting in increasing prevalence of trypanosomiasis in both human and animal hosts, and the spread of rHAT into previously unaffected areas. Risk factors include human proximity to the large wildlife reservoir in the South Luangwa National Park to the north-west [2], and ever-increasing livestock and human density on the plateau. Little is known concerning tsetse-trypanosome-human interaction in the region. Therefore, the ABM has the potential to enable exploration of contact risk within communities. Furthermore, with climate changes expected to occur in the near future, such as reduced annual rainfall, increased storm events and increased temperature [57][58], it is becoming increasingly important to understand how climate factors can affect tsetse populations, particularly in areas such as this, where increases in temperature could see the tsetse habitat spreading further up the valley to more populous areas.

### Adding seasonality–The exploration of climatic drivers

This paper describes a new, seasonally sensitive ABM for rHAT/animal African trypanosomiasis (AAT), based on an earlier, non-seasonal model that was constructed using data derived from a detailed rHAT, AAT, and *G*. *m*. *morsitans* ecological survey, undertaken in 2013, in Eastern Province, Zambia [27]. Due to the fine spatial and temporal scales used to model the system, and the number of mechanisms incorporated (e.g., tsetse reproduction, tsetse feeding, human agent movements using real-world routines and pathfinding techniques [59]), the model was complex and its data inputs were numerous. As a result, only new data and modifications to the original model are described here. A detailed description of the original, non-seasonal model framework, and the data used to construct it, can be found in [27].

#### Temperature data

**Fig 3:**
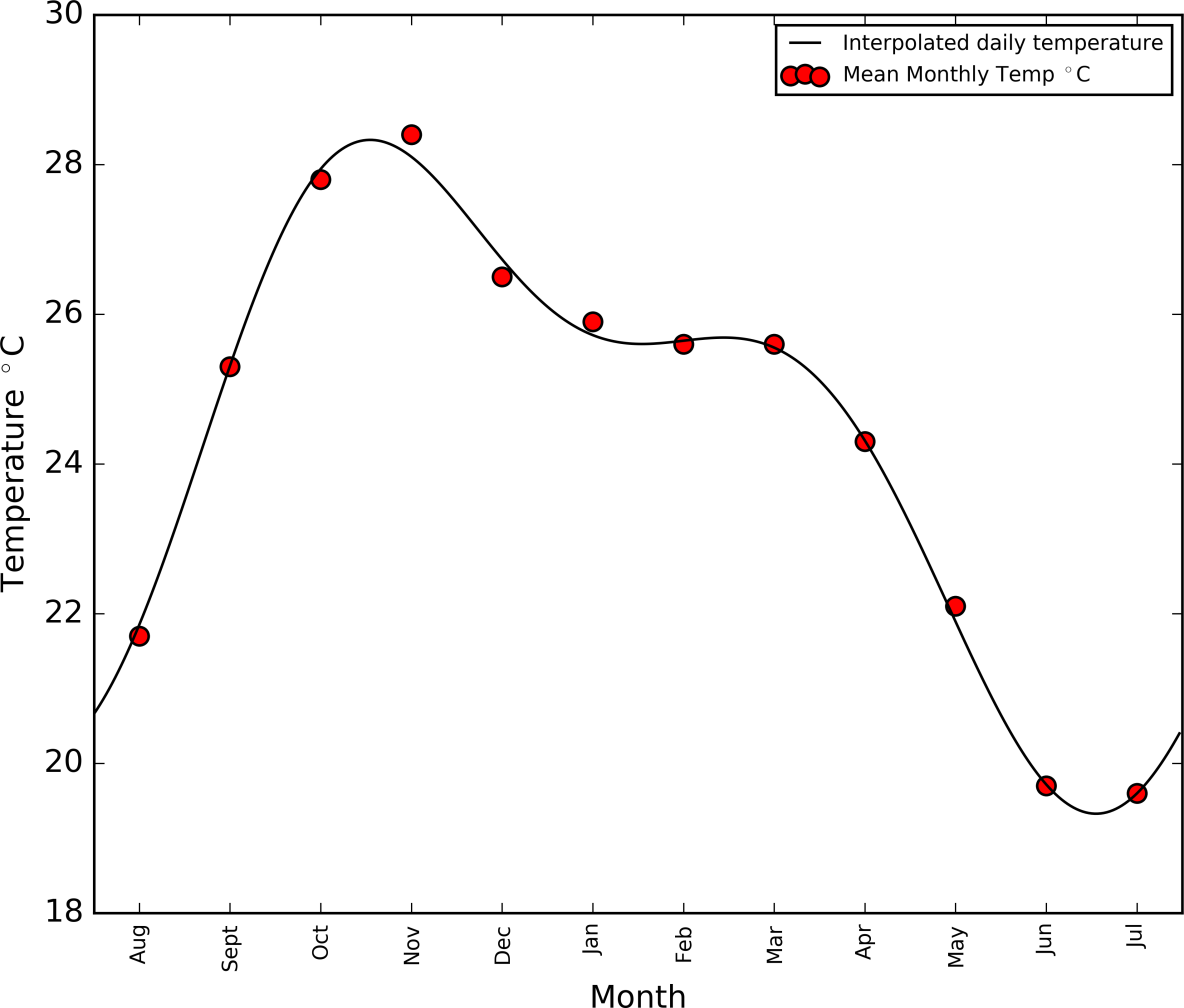
*Interpolation of mean monthly temperature data using sum of sines technique (*r^*2*^ *= 0*.*97)*.

Annual mean temperature data collected at Mfuwe airport are presented in Fig 2. Fig 3 shows the interpolation of these data into daily temperatures using a four-term, sum of sines method, produced using Matlab’s curve-fitting tool. This method produced the best fit to the mean monthly data (*r*^2^ = 0.97), while also producing limits which join when wrapped, allowing multiple years to be considered. Future mention of temperature in this section refers to the daily temperatures derived from this curve. The temperature model is presented starting in August as this reflects the month in which the present simulation commences, chosen as the midpoint between the tsetse surveys used to estimate the tsetse population (June and November–see [27]).

#### Seasonal variation in oviposition

**Fig 4:**
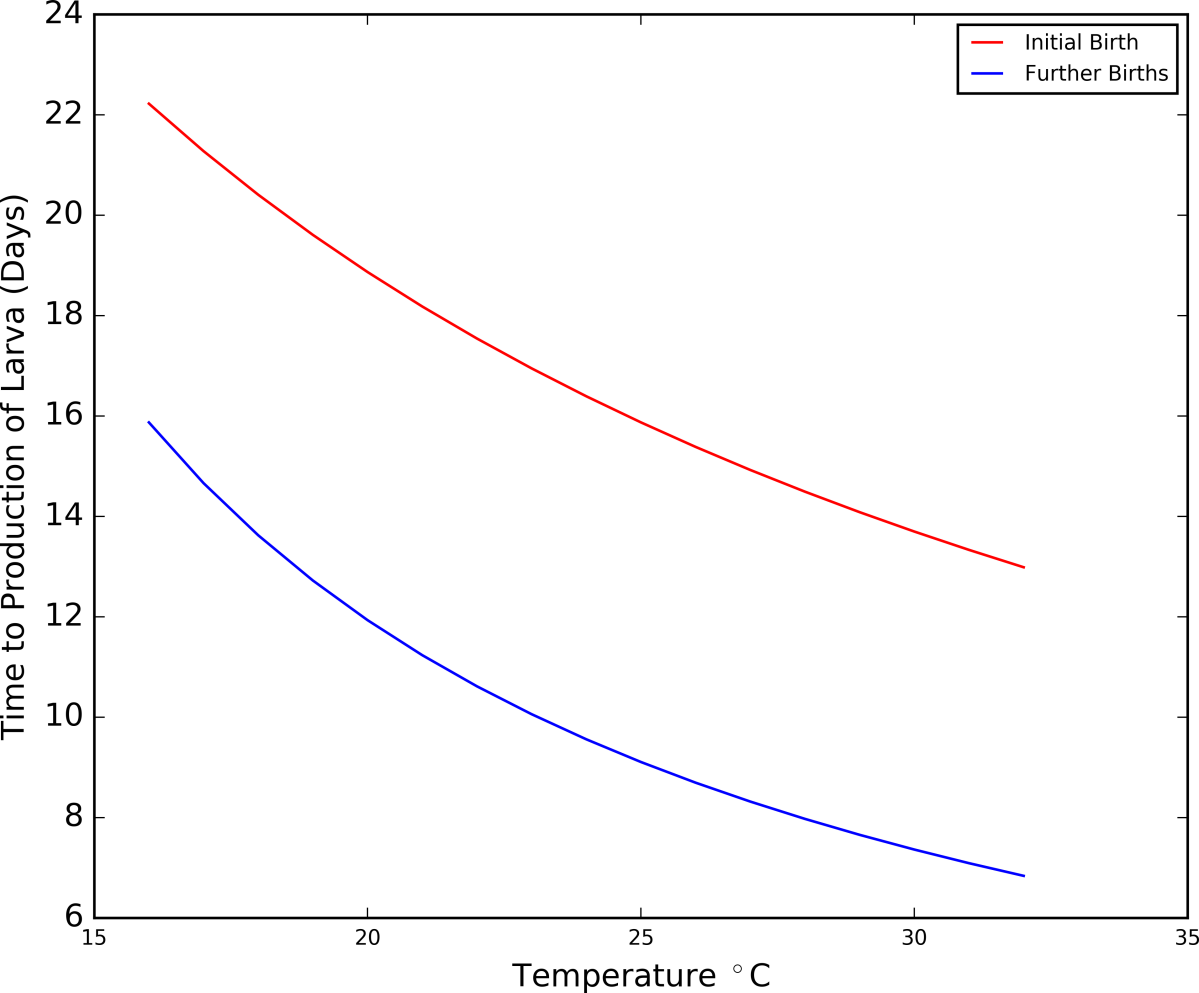
*Tsetse reproduction intervals in response to temperature (produced using data from* [41]*)*.

The non-seasonal model used the expectation that a female tsetse would give birth 18 days after mating, and every 10 days thereafter for the duration of their life [60]. However, data collected in Zimbabwe in 1994 suggest that in warmer conditions (~30°C), the time taken to produce a first pupa can be as low as 15 days [40], with extrapolations of the data suggesting that subsequent offspring could be produced at an interval of 16 days at 16°C, and 7 days at 31°C [41] (see Fig 4) using Eq 1 [41]:

$$Birth\;Interval=\frac{1}{k_{1}+k_{2}(T-24)},$$ Eq 1

Where: *T* = temperature, *k*_1_ = 0.061 and *k*_2_ = 0.0020 for the initial birth interval, and *k*_1_ = 0.1046 and *k*_2_ = 0.0052 for further births.

Although extrapolation should be treated with caution, the tsetse species under investigation, along with the vegetation, are consistent in both studies. Furthermore, the temperatures in the region of the present study are largely in the range of temperatures for which field experimental data were collected to produce these curves (22°C to 30°C) and, as a result, are used to dictate birthing intervals in the current simulation.

### Temperature and pupal duration

**Fig 5:**
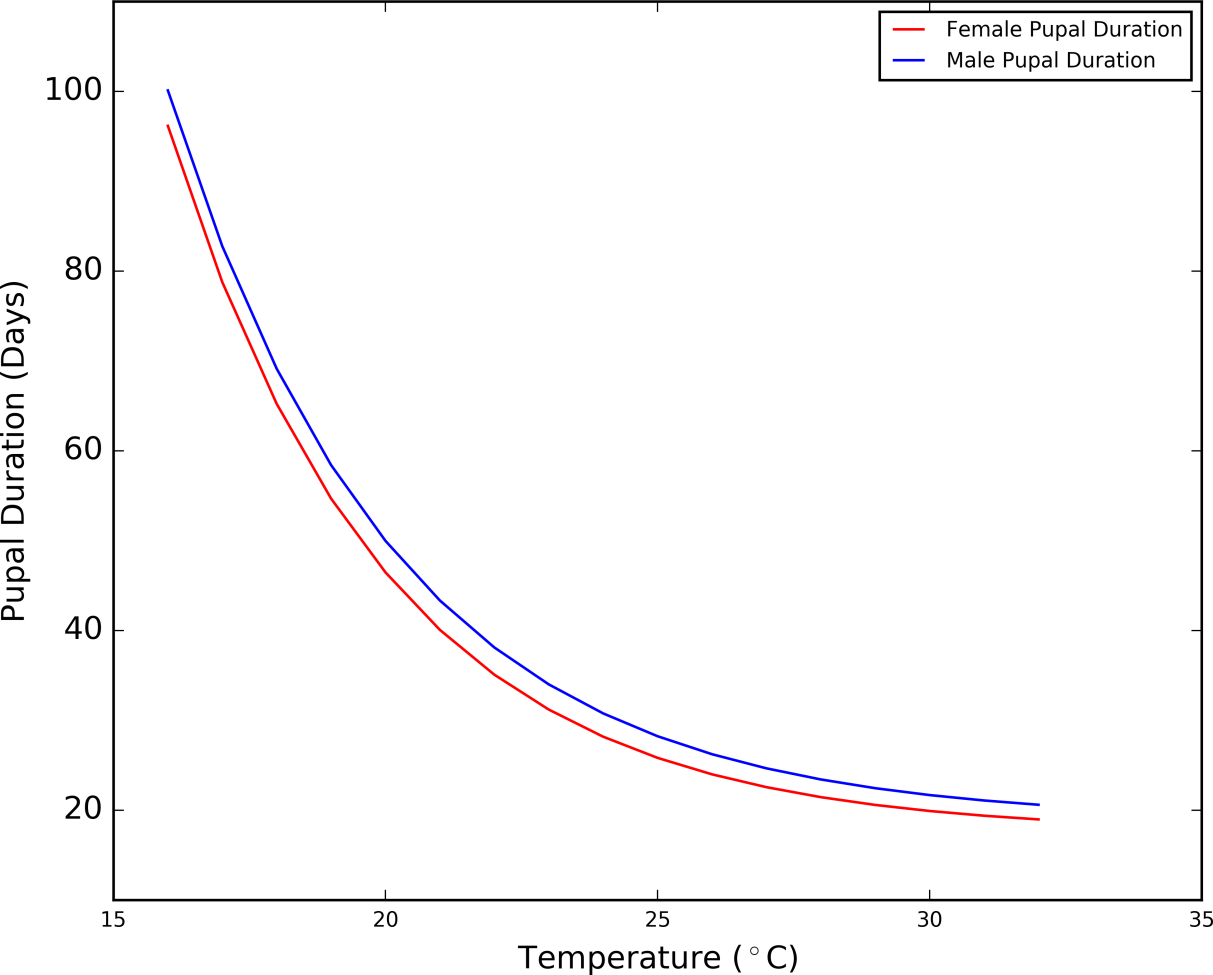
*Tsetse pupal intervals in response to temperature (produced using data from* [41]*)*.

The previous iteration of the model included a longer pupal duration in males than in females, as suggested in the literature (e.g. [37,60]), and so for each larva deposited during the simulation, a 35 and 30 day pupal period was included for males and females, respectively, represented as a period of inactivity. However, pupation is known to be temperature sensitive with pupal periods decreasing with increasing temperature, a relationship observed by Phelps and Burrow’s laboratory experiments at constant temperatures [37]. Hargrove [41] utilised the data to present a near perfect fit for pupal duration at temperatures between 16°C and 32°C (r^2^ = 0.998) (see Fig 5), represented by Eq 2:

$$r=\frac{k_{3}}{1+e^{\left(a+bt\right)}},$$

$$pupal\;duration=\frac{1}{r},$$ Eq 2

Where: *t* = temperature, for males: *a* = 5.3, *b* = -0.24 and *k*_*3*_ = 0.053 and for females: *a* = 5.5, *b* = -0.25 and *k*_3_ = 0.057.

Given the excellent fit to the data and the large variation in pupal periods expected within the temperature range found in the study area (19°C = ~60 days, 28°C = ~20 days), variation in pupal duration with temperature is clearly an important factor to incorporate in the model.

#### Climatic drivers for pupal mortality

Due to the difficulty in finding pupae in the field, population estimates, and ultimately estimates of pupal death rates are rare [41]. Although observations suggest temperature may not affect pupal mortality until extremes are reached (>35°C) [39], pupal mortality in tsetse flies is shown to vary seasonally [61]. Similar to Vale and Torr’s [29] climate independent tsetse model, the previous iteration of this ABM assumed a pupal mortality rate of 26%, after field observations by Jackson [62]. However, as a result of the implementation of a variable pupal duration in the current version, it seems appropriate to apply pupal mortality rate on a daily basis. While the 1% daily pupal mortality rate field estimates presented by Rogers and Randolf [63] were used as a starting point here, Ackley and Hargrove [61] found that mortality rates in immature fly stages peak before and after the rainy season, with model output for *G*. *pallidipes* reporting a peak mortality rate of 0.12 during March in Rekomitjie, Zimbabwe. To capture this apparent variability, the pattern presented was adapted and applied to the current investigation. Modifications were required as the pattern of mortality presented by Ackley and Hargrove considers pupae and immature flies together, while the model in this study models pupae separately–resulting in a risk of double counting mortality in immature flies. In addition, the species of tsetse fly under investigation here is *G*. *m*. *morsitans* rather than *G*. *pallidipes*—*G*. *pallidipes* pupae are found to show greater susceptibility to extreme temperatures (e.g. [64]). As a result of these factors, simply applying the same mortality rates to the current investigation would overestimate pupal mortality, with tests showing that the values of daily mortality reported by Ackley and Hargrove needed to be scaled to 60% to provide stability in the current investigation. Mortality rates were applied such that each day, each pupa had a probability of dying based on these scaled mortality rates.

#### Combining age and temperature dependent mortalities

**Fig 6:**
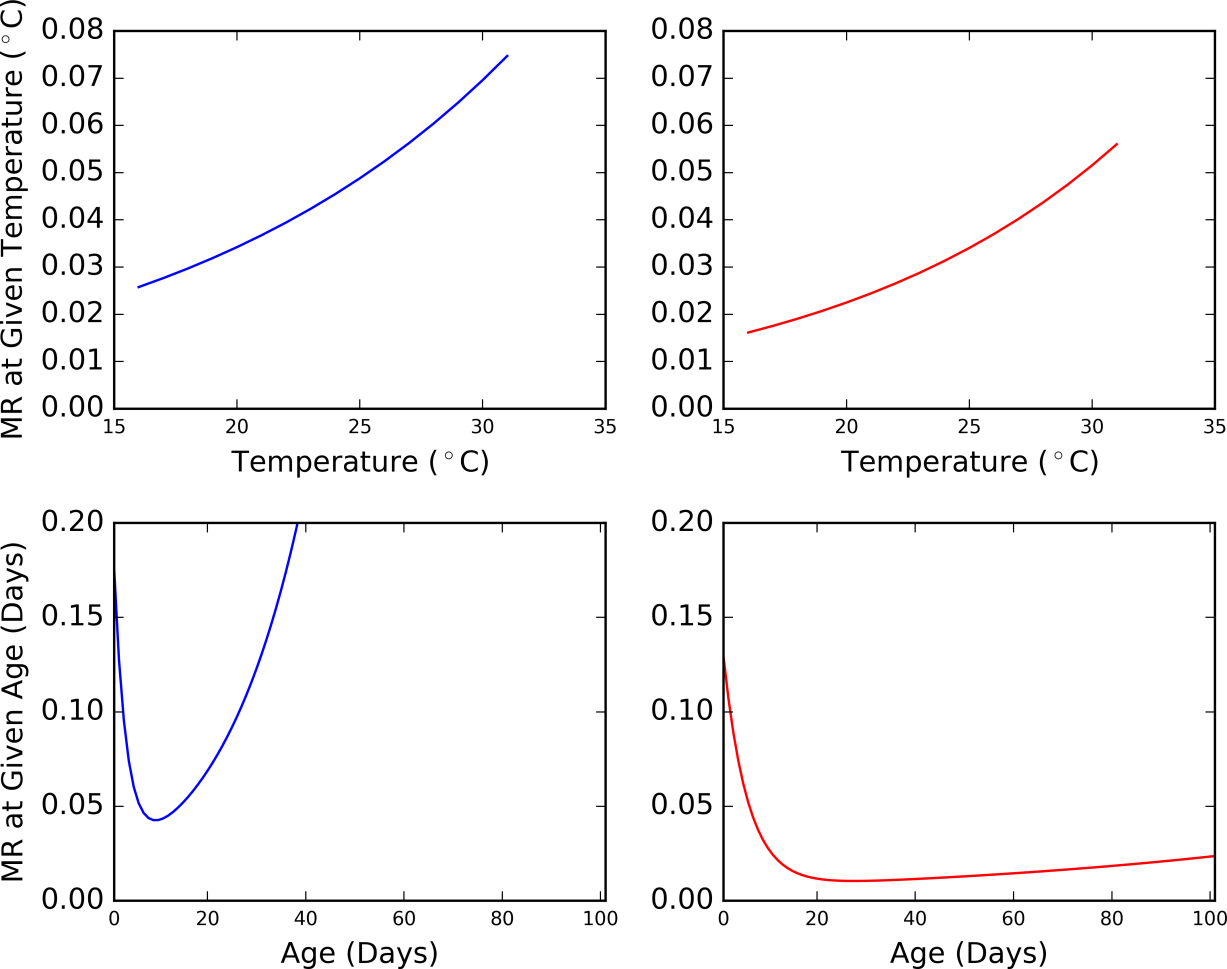
*The upper two plots present an observed relationship between tsetse mortality rate (MR) and temperature (produced using data from* [41]*)*. ***The lower two plots illustrate a modelled relationship between tsetse age and mortality rate (produced using data from*** [65]***)*. *Blue = male tsetse*. *Red = female tsetse***.

The first iteration of the model included a tsetse death function to deal with mortality not captured by starvation of individual tsetse, or pupal mortality. Represented as a scaled-down version of Hargrove’s age-dependent mortality model [65], no climatic impact was incorporated. To ensure the tsetse agents are sensitive to temperature in the current iteration of the model, the previous age-dependent mortality rate (Eq 3) [65] was adapted to reflect the relationship reported by Hargrove in Eq 4 [41], a modelled estimate of daily mortality rate in response to temperature based on mark-recapture data collected on Antelope Island in Zimbabwe in 1980 and 1981. Eqs 3 and 4 are visualised for both male and female tsetse in Fig 6.

$$\lambda_{1}(t)=k_{4}(k_{5}e^{-k_{5}a(t)}+k_{6}e^{k_{6}a(t)}),$$ Eq 3

Where: *t* is a point in time, *a(t)* is fly age at time *t*, and *k*_*4*_, *k*_*5*_, *k*_*6*_ are gender-specific constants (Male: *k*_*4*_ = 0.389, *k*_*5*_ = 0.395, *k*_*6*_ = 0.0583 and female: *k*_*4*_ = 0.605, *k*_*5*_ = 0.201, *k*_*6*_ = 0.0119) [65].

$$\lambda_{2}(t)=\frac{e^{-k_{7}+(k_{8}T(t))}}{100.0},$$ Eq 4

Where: *T*(*t*) is the temperature at time *t*, and *k*_7_, *k*_8_ are gender-specific constants (Male: *k*_7_ = 0.19, *k*_8_ = 0.071 and female: *k*_7_ = 0.85, *k*_8_ = 0.083) [41].

Both Eqs 3 and 4 are hazard functions, which define the likelihood that something will survive to a certain point in time based on its survival at an earlier time, each taking a different factor into account. Examples of combining hazard functions are well recorded in reliability engineering, where “failure” is used in place of “hazard” or “mortality”, and the failure rate of a series system is calculated as the sum of the failure rates of its components [66]. While more complicated methods are required to calculate failure rates of parallel and combined systems, in complex systems, where relationships between two components cannot be defined easily, the system’s failure rate is pessimistically taken to be the sum of the individual failure rates of its components. Since the relationship between age and temperature on the tsetse mortality rates are not well defined, the latter method has been implemented for this study (Eq 5).

$$\Lambda (t)=\sum_{i=1}^{n}\lambda_{i}(t),$$ Eq 5

where, *λ*_*i*_ (*t*) is the hazard rate for ‘component’ *i*.

While the temperature-dependent mortality rate (*λ*_2_(*t*)) will have been determined across a range of fly ages, the age-dependent mortality rate (*λ*_1_(*t*)) was determined during a relatively small window in which the temperature was likely to be constant. To avoid ‘double counting’ the mortality rate due to temperature, the age-dependent mortality rate was decoupled from temperature to remove any associated effects, via the new function in Eq 6. While this is relatively crude, this was used as a starting point in the absence of a more detailed understanding of the relationship between temperature and age in the mortality of the tsetse fly.

$$\lambda_{1}(t)=k_{4}(k_{5}e^{-k_{5}a(t)}+k_{6}e^{k_{6}a(t)})-\frac{e^{-k_{7}+(k_{8}T_{c})}}{100.0},$$ Eq 6

where, *T*_*c*_ is a constant temperature, which could be considered as the ‘base’ temperature.

**Fig 7:**
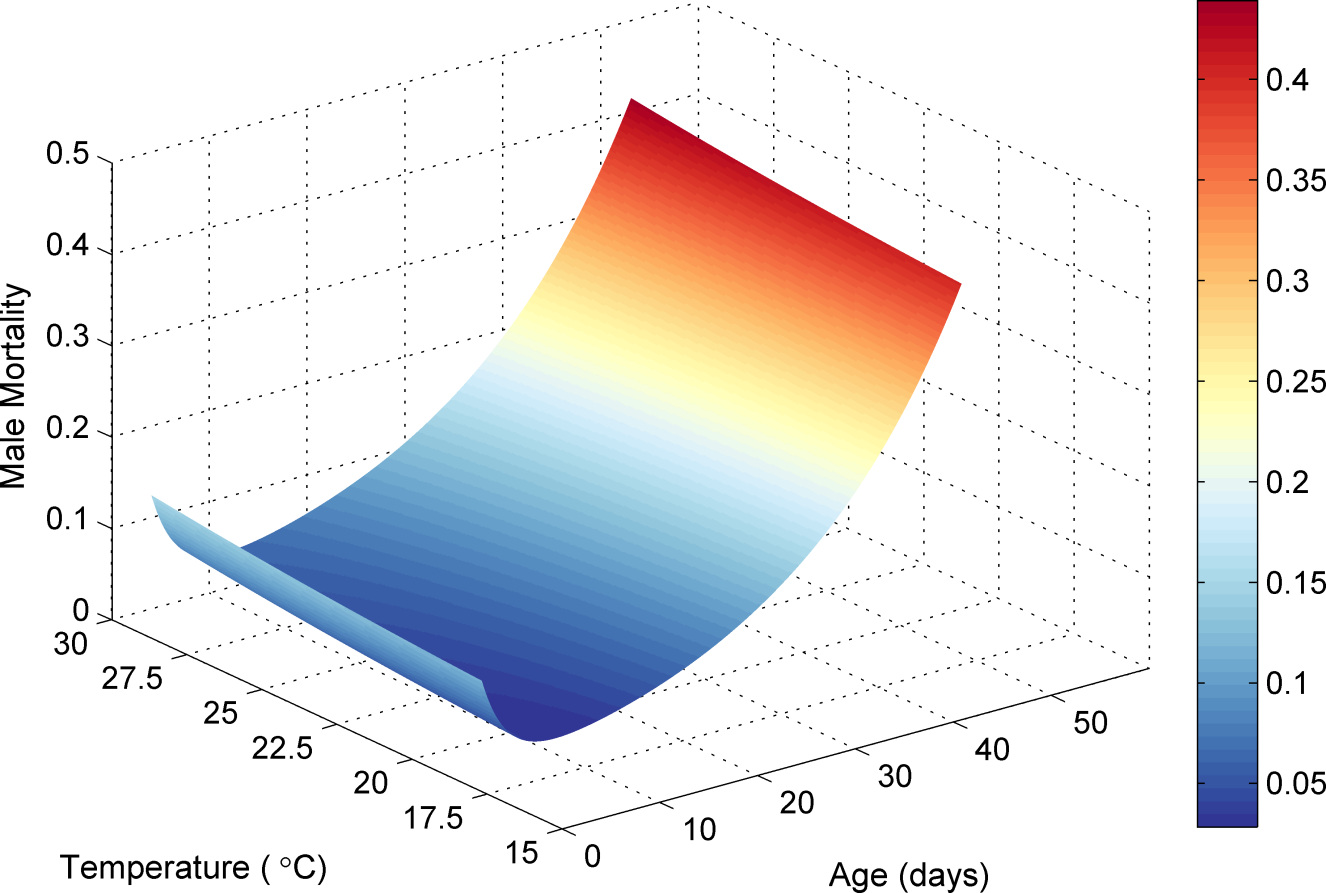
Male tsetse mortality rate dependent on age and temperature.

**Fig 8:**
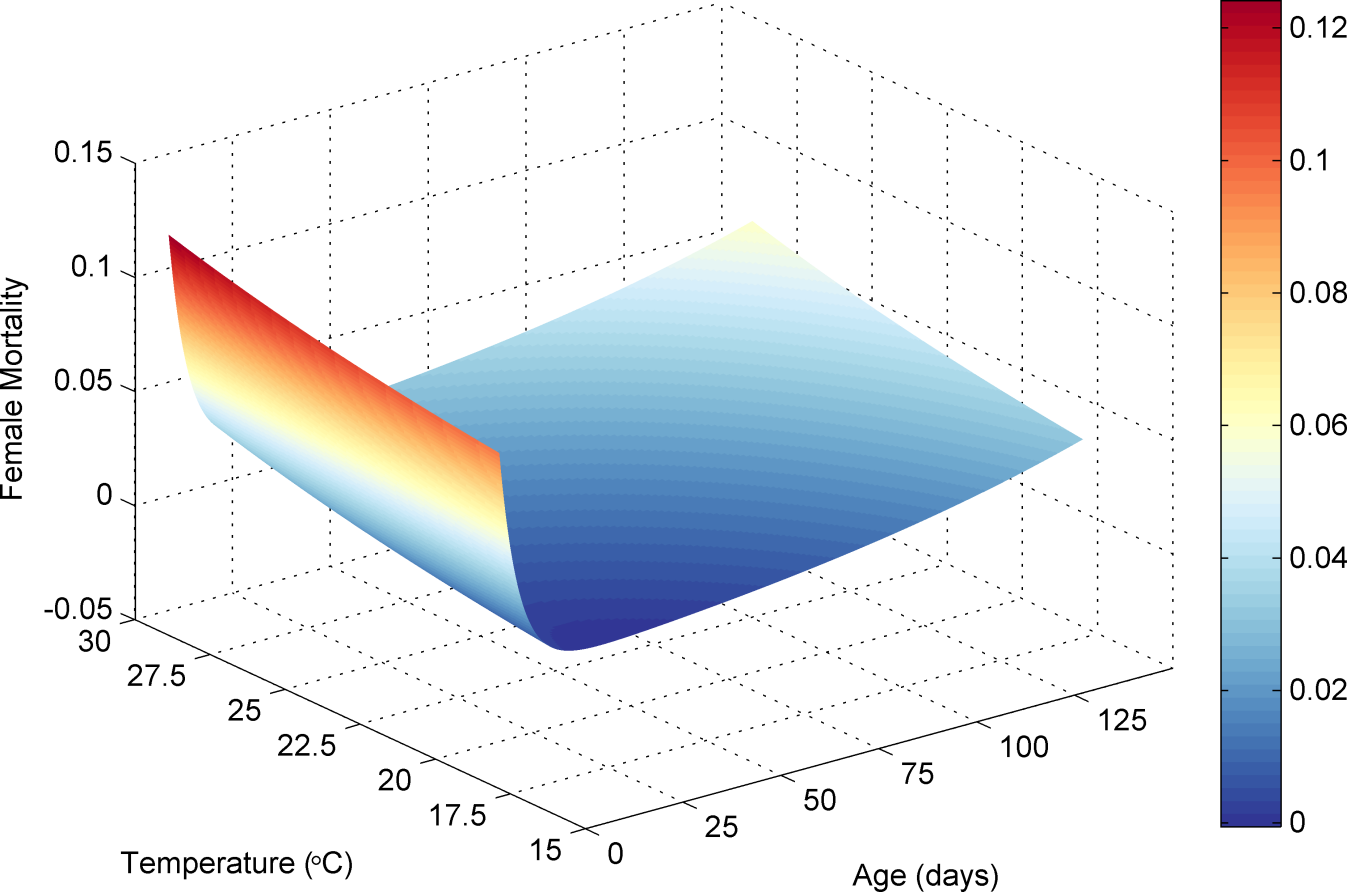
Female tsetse mortality rate dependent on age and temperature.

Using this equation and the constant values reported previously, the daily mortality rate of a tsetse fly at a given age and temperature can be visualised as in Fig 7 for males and Fig 8 for females.

### ABM framework

The previous, non-seasonal ABM provided the majority of the methods and data used in the current version of the simulation, and so readers are referred to [27] for greater detail and only a summary is provided here. Census data were used to locate and initialise the human and animal populations living in the households shown in Fig 1. A sample of resource-seeking routines sorted by gender and age was taken in the field (see supplementary information of [27]), and a set of plausible paths from each village to each resource was created using a pre-processing A* pathfinding technique [59]. For tsetse, an estimate of the total apparent population size, density and distribution was provided. Four agent types were included in the ABM, together with an areal representation of wildlife. Humans, cattle, other domestic animals and tsetse used in the ABM were constructed as four separate classes, with populations modelled 1:1 with the data collected in the census (e.g. 16,024 human agents) and the estimated tsetse population discussed previously. Each class had its own initial information and storage structures for events that occurred through the simulation. The ABM was written in Python 2.7 using an object-oriented framework, and run on the Lancaster University High End Computing (HEC) Cluster, with all spatial data being processed using Quantum GIS 1.8.0.

The subsequent sections draw attention to any modifications between the original, non-seasonal modelling framework and the new ABM model, while also describing how the climatic drivers affecting the tsetse population were incorporated into the model.

### Temporal resolution and wildlife feeds

The initial iteration of the model was split into 2,400 time-step (or tick) days, as the more frequent the tick, the smaller the jumps made by agents as the simulation updates, and the less chance of missing potential interactions. However, this method was restrictive in terms of memory usage and CPU time required to run just six months of the simulation. Further tests were carried out to establish how coarse the temporal resolution could be made before the number of simulated domestic host-vector contacts was reduced, and a greater daily probability of wildlife feed was required to maintain the tsetse population levels. It was established that 600 ticks per day (2.4 minutes per tick) allowed the simulation to progress with no obvious effect on human, cattle and other domestic animal bite numbers, while requiring a very similar daily wildlife bite probability to produce a stable tsetse population (37% chance per day of a hungry tsetse taking a wildlife bite, compared with 35% in the previous version). As a result, 600 ticks per day were used to produce the results of this investigation, which required approximately 4.5 GB of RAM per simulation run on the high performance machine, and 24 hours of CPU time per simulated year.

### Climatic driver additions

To capture the effect of seasonality on the tsetse fly population, daily temperature was calculated every 24 hours using the interpolation method discussed previously, and set as a global variable for the simulation.

### Tsetse births

For each female, once mated, the number of days since mating was compared with the birth interval calculated using Eq 1 and the daily temperature. If and when the number of days since mating exceeded the interval calculated on a given day, a pupa was deposited. A count of the number of days since mating was replaced with a count of the number of days since last offspring, and Eq 1 was used again on a daily basis (using the alternative constants for further births), until another birth occurred. This process was repeated for the duration of a female tsetse fly’s lifespan. There was an equal chance of each tsetse offspring being male or female, and each pupa was deposited in a bush area in which the female tsetse rested during the previous night.

### Pupal duration and death

A rolling average of the temperature that each pupa has experienced since birth was calculated and attributed to each individual. This temperature was used to determine each individual’s pupal duration, given that if a pupa’s age exceeded the pupal duration calculated using Eq 2, the pupa would emerge as a teneral fly. It was considered important to use a rolling average of temperature here as the length of a pupal period can span months with quite different temperatures. As described previously, rather than a single probability used to decide whether a pupa would die during its entire pupal period, a variable daily probability of pupal death was included, increased in some months to account for losses observed in the rainy season. Should the probability be exceeded for a pupa, that tsetse was removed from the simulation.

### Teneral and mature tsetse death

Death could result from pupal mortality, starvation, or if a tsetse fly exceeded the daily mortality rate calculated by sex, age and temperature (Eq 6, Figs 7 and 8). The mortality rate was calculated individually for each teneral and mature fly, and if the probability was exceeded, the tsetse was removed from the simulation. Starvation occurred if a tsetse tried and failed to feed before a given period of time had elapsed. The starvation element was more strict for teneral flies (3 days instead of 5 days) highlighting their increased vulnerability and reduced flight strength. In the previous version of the simulation, 75 teneral files were added to the simulation for the first 35 days to account for pupae deposited prior to the start of the simulation. As this version of the simulation started in August, and the simulated climate quickly became hostile for teneral flies as temperature increased, 500 teneral tsetse were required per day for the first 45 days, which is representative of average simulated pupal maturation rates during September as the simulation progressed (see Results). In the original model, in the absence of climatic factors, a scaling factor for adult fly mortality was required to offset fly starvation within the simulation. This value was set at 55%. Although this scaling factor is still required in this iteration of the model due to the same starvation element, the incorporation of temperature dependent mortality, and more detailed mechanisms for modelling pupae, has reduced the required level of scaling to 80%

To allow the model to initialise and stabilise, the simulation was run for a year before the results for this paper were produced, allowing a ‘burn-in’ period. For example, the results presented below are representative of years 2–4 of the simulation. 100 repeat simulations were used to produce the results presented here.

## Results

### Tsetse population

**Fig 9:**
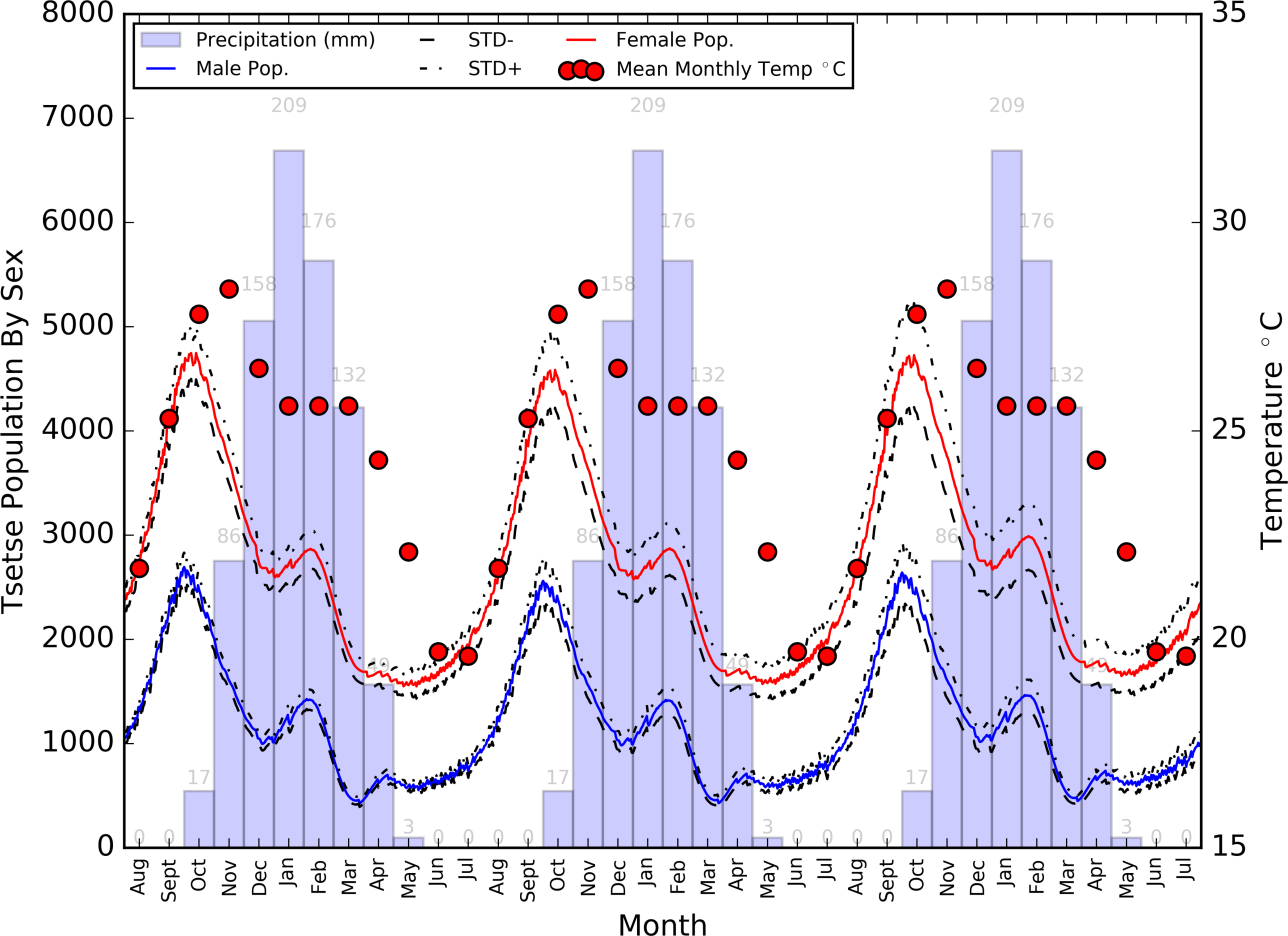
Average daily population size of adult tsetse flies by sex (+/- standard deviation), overlain on mean monthly temperature and precipitation for the three year simulation period.

At the end of the three year simulation, a relatively stable population record was observed in both the male and female tsetse populations, with both exhibiting a double peak in response to the climatic driver (Fig 9).

**Fig 10:**
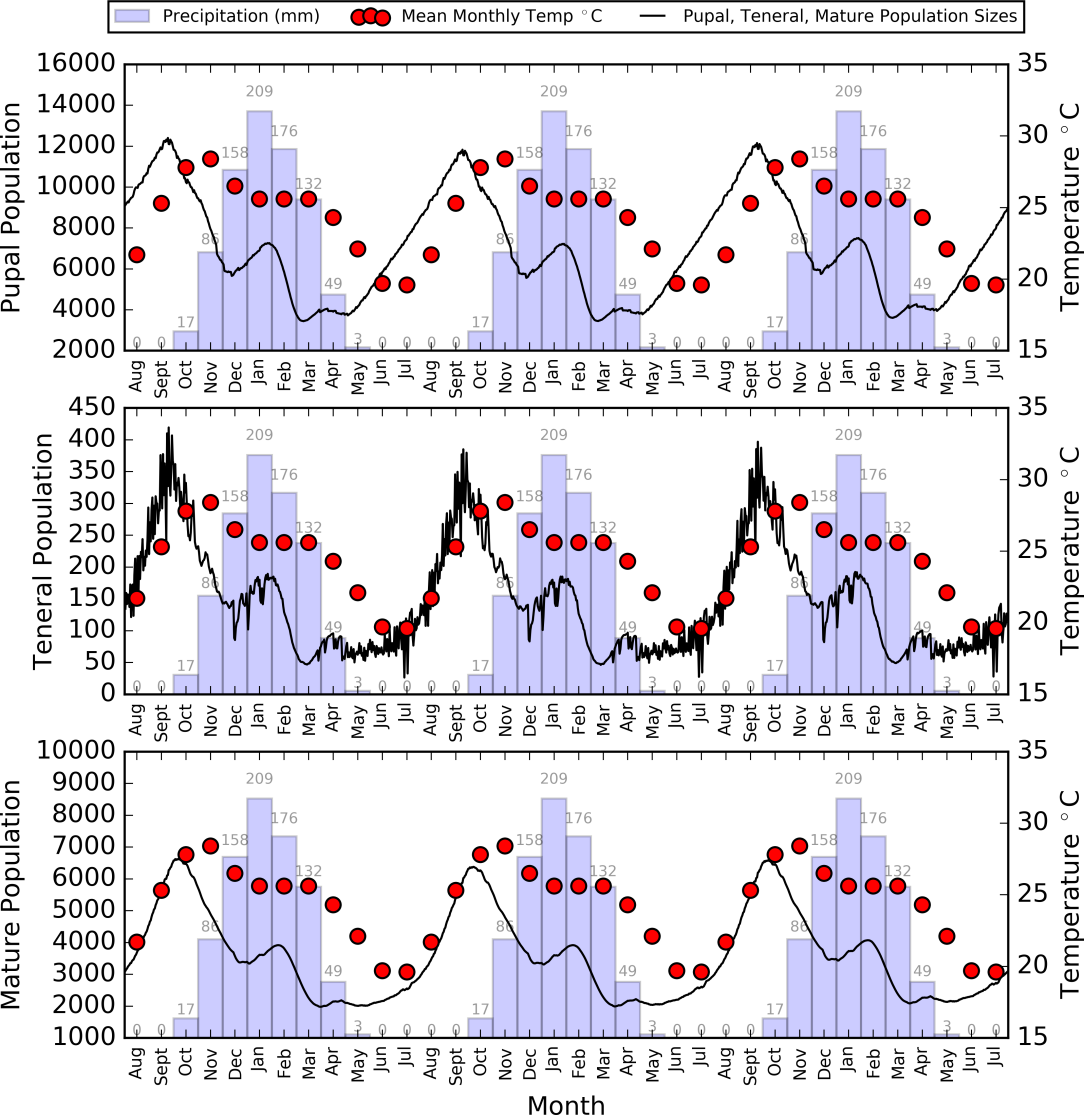
Average daily population size of tsetse flies at different life stages, overlain on mean monthly temperature and precipitation for the three year simulation period.

**Fig 11:**
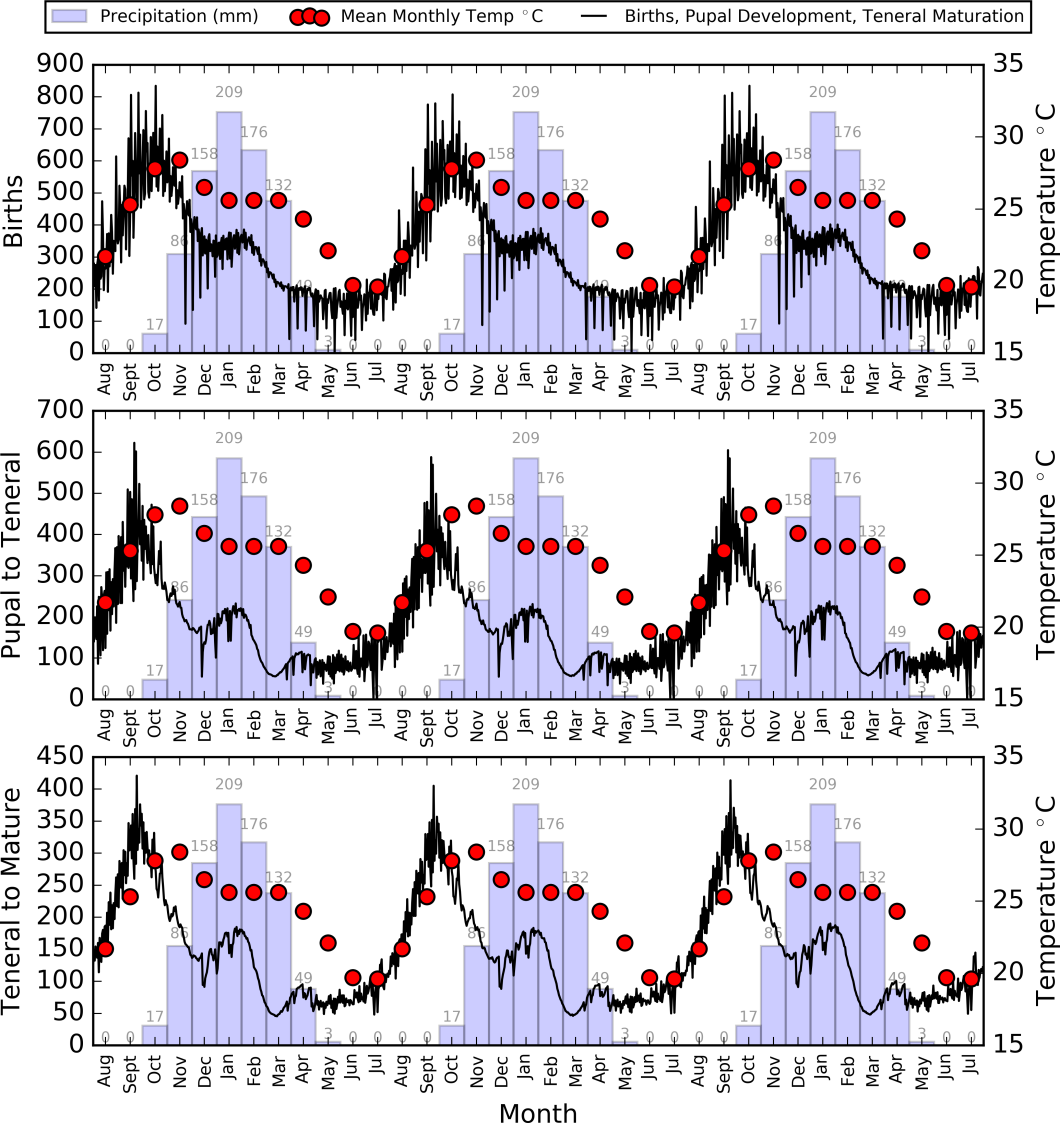
Average daily numbers of population transitions within the tsetse population, including initial birth, the development from a pupa to teneral fly, and the development from teneral to mature fly on first feed.

Each year, until peak temperature was reached in October and November, the population slowly increased, with each gender’s population size increasing by approximately 2000 flies. Such population increases during this hot and dry season could be attributable to the absence of a boosted pupal mortality which is observable during the rainy season [61], with increasing temperatures having a greater impact in reducing pupal duration and the period between births, than increasing tsetse teneral and mature tsetse mortality. During the rainy season (November-April), this population gradually fell to an annual low, a result of peaks in pupal mortality at the start of the rainy season, and high temperatures causing increased mortality in the annual peak population of teneral flies (see Fig 10) (now emerged after a high period of births discussed previously—birth numbers can be seen in Fig 11).

During this period, with a reduced number of pupae to develop, and teneral tsetse to mature and start reproducing, the higher temperatures no longer aided a growth in population as there were fewer pupal maturations and birth rates to ‘accelerate’ (Fig 11) At the end of the rainy season, the tsetse population gained a small boost due to a plateau in temperature, and the drop in population slowed through the cool and dry season (May to July), although recovery did not start during this period as temperatures were too low to aid rapid repopulation of the tsetse, and the pupal population was still recovering (Fig 10).

**Fig 12:**
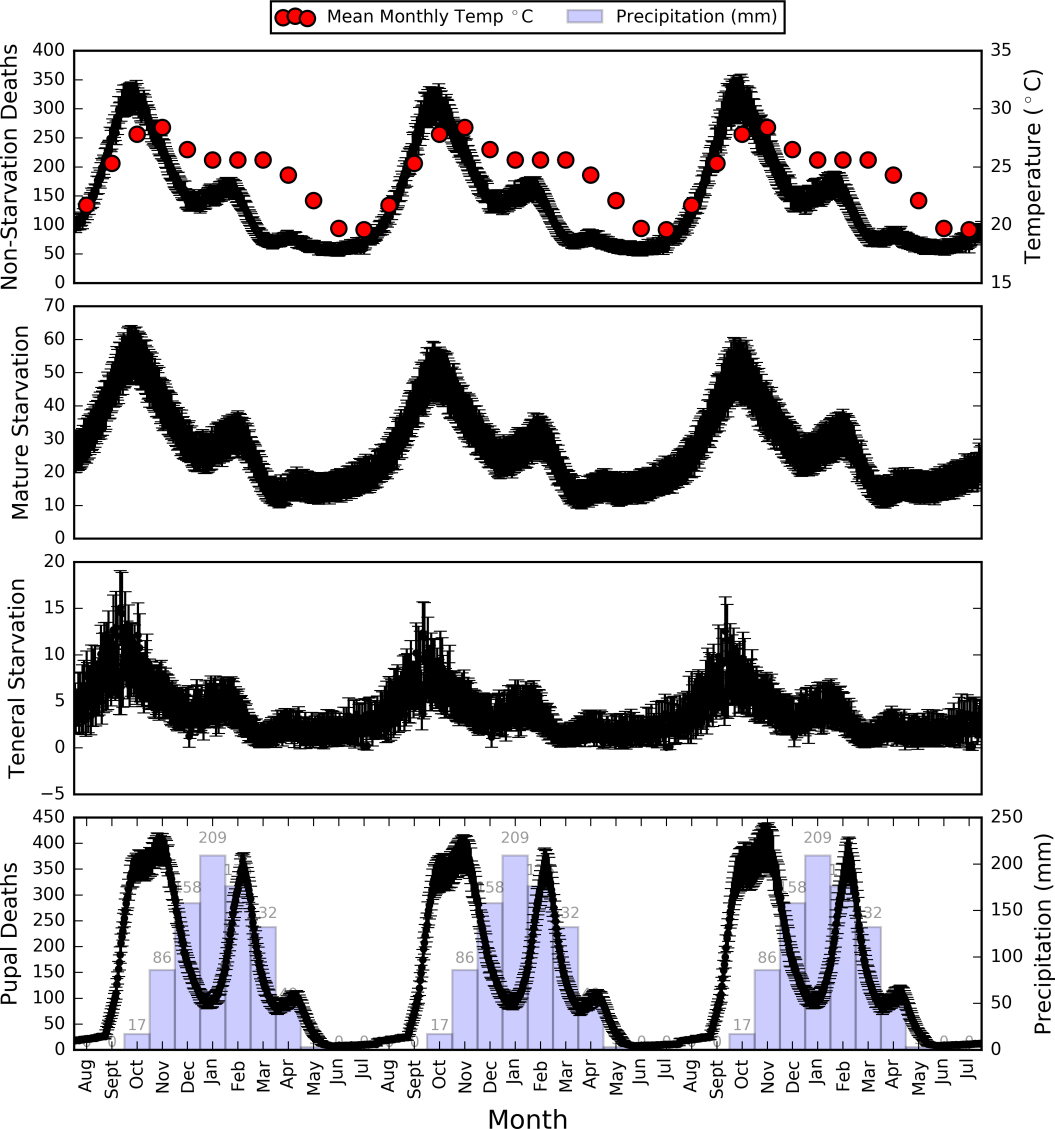
Average daily deaths by class for the tsetse population, including starvation of both teneral and mature flies, pupal mortality, and other deaths attributable to the age and temperature dependent mortality model discussed previously. Error bars represent the standard deviation.

Fig 12 presents the different possible modes of tsetse death included in the model, and how the rates varied as the simulation progressed. Non-starvation death represented the deaths attributable to the age-temperature dependent mortality model defined by Eq 6, and was consistently responsible for the largest number of daily deaths, peaking in the period of highest temperature with approximately 350 deaths per day. Unsurprisingly, given its temperature dependency, the mortality shape closely aligned to mean monthly temperature, except for a period in February and March after the pupal population was reduced by a period of high pupal mortality during the rainy season, resulting in a reduced teneral population and, therefore, fewer adult deaths. Deaths due to starvation followed closely the general pattern of population size, with teneral starvations being particularly low–likely a result of the low daily teneral population size (ranges between 100 and 400 –Fig 10) and the teneral tsetse population having the highest age-temperature dependent mortality rate. Using the Ackley and Hargrove model [61] for pupal mortality produced peaks prior to the rainy season and, to a lesser extent, after the wettest months (Fig 12).

The ratio of pupae to mature tsetse was approximately 2:1 at any given time, with the mature to teneral population ranging between 15:1 at the peak of population size and 25:1 when population sizes were generally lower.

### rHAT transmission

Across the three year simulation, the approximate incidence rate for human and cattle rHAT infections was 0.355 per 1000 person-years (SE = 0.013), and 0.281 per 1000 cattle-years (SE = 0.025). There were 11 human infections each year on average (i.e. per year, per run), and 2 cattle infections.

**Fig 13:**
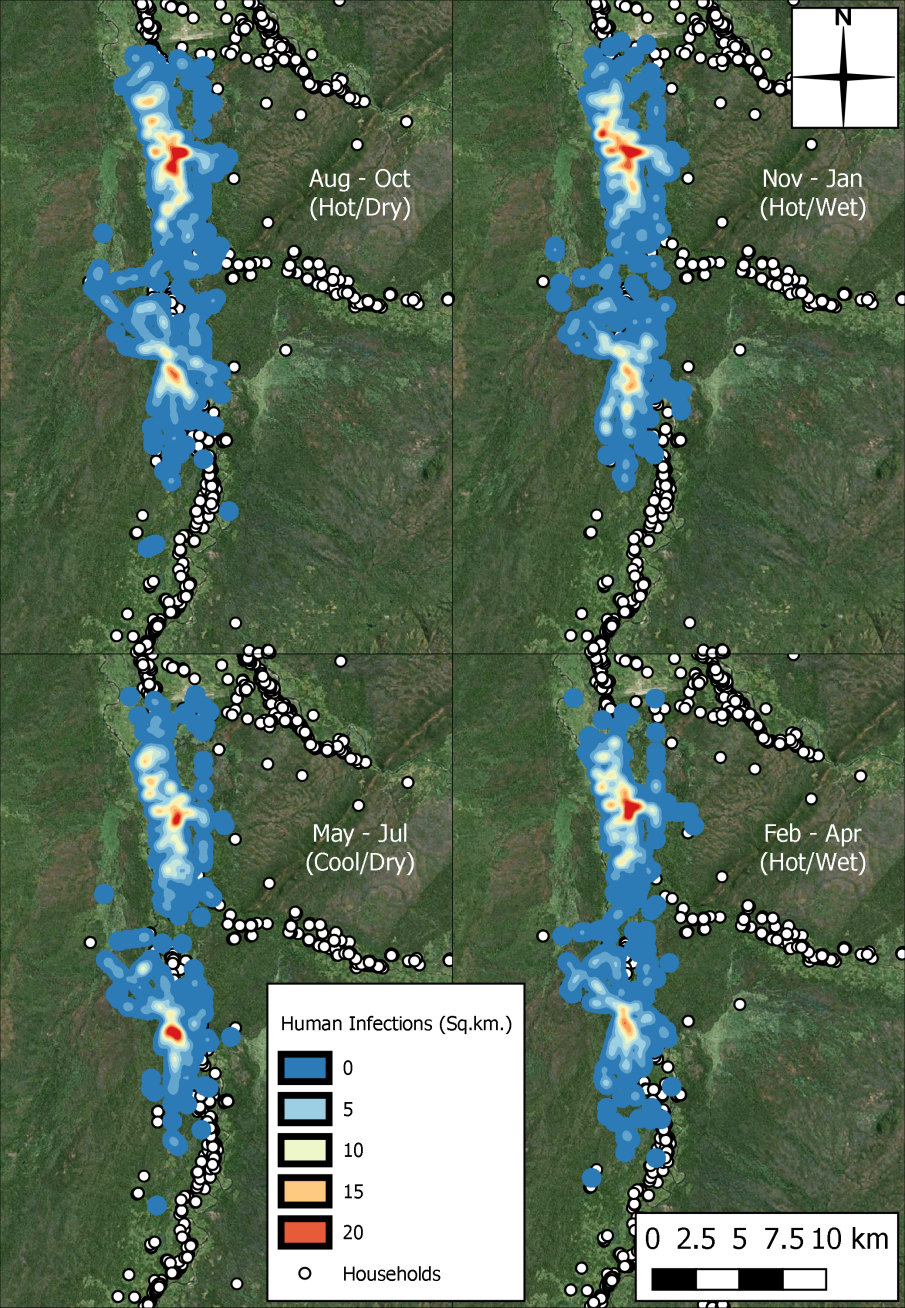
Heat surface representing aggregate number of infections across the 100 repeat simulations, by season, with pixel values taking the units of infections per square kilometre.

Fig 13 illustrates how these infections clustered spatially and by season. The aggregate number of infections across all years and each of the 100 repeats was used to produce this heat map due to the low infection numbers. There was not much spatial variability through the seasons despite the variation in tsetse population size.

However, the number of infections reduced during the second half of the rainy season with the lowest density of infections observed during the cool and dry months. Two hotspots are visible in each of the seasons, each with elongated elements suggesting that frequently used paths were sources of interaction between vector and human host. This is possibly most visible in the north as east-to-west movement here could represent movement between villages and the river, a hypothesis which is given support by observations of infection by activity (Table 1) which suggest that in each season, water collection accounted for approximately 25% of human infections, second only to school trips which accounted for 49% to 51% of infections. No human infections were acquired whilst watering or grazing cattle, while the third highest number of infections occurred when farming. There was little variation in infections by activity between the seasons.

**Table 1:** Average proportion of human infections attributable to each activity, for each season, represented as a percentage.

	Total (3 years)		Hot, Dry Season		Hot, Wet Season		Cool, Dry Season	
State	*Average*	*SE*	*Average*	*SE*	*Average*	*SE*	*Average*	*SE*
*School*	**50.0%**	1.2%	**49.5%**	2.1%	**50.1%**	1.7%	**51.1%**	2.8%
*Resting*	**3.2%**	0.3%	**3.7%**	0.8%	**3.4%**	0.5%	**2.4%**	0.6%
*Market*	**0.1%**	0.1%	**0.4%**	0.2%	**0.1%**	0.1%	**0.0%**	0.0%
*Firewood*	**5.8%**	0.5%	**5.9%**	1.0%	**6.0%**	0.7%	**3.8%**	0.7%
*Farm*	**15.3%**	0.8%	**14.8%**	1.5%	**16.0%**	1.1%	**16.9%**	2.2%
*Water*	**25.5%**	1.2%	**25.7%**	1.9%	**24.4%**	1.6%	**25.8%**	2.3%
*Graze Cattle*	**0.0%**	0.0%	**0.0%**	0.0%	**0.0%**	0.0%	**0.0%**	0.0%
*Water Cattle*	**0.0%**	0.0%	**0.0%**	0.0%	**0.0%**	0.0%	**0.0%**	0.0%

With the observed high proportion of infections coming from school trips, it is unsurprising that 5–10 year olds and 10–18 year olds had the highest infections rates (Table 2). Infection rates were generally lower in the cool and dry season, peaking in the hot and dry season.

**Table 2:** Approximate incidence rates by season for different age groups.

	Total (3 years)		Hot, Dry Season		Hot, Wet Season		Cool, Dry Season	
Age	*Average*	*SE*	*Average*	*SE*	*Average*	*SE*	*Average*	*SE*
*age1-5*	**0.0241**	0.0037	**0.0231**	0.0072	**0.0262**	0.0058	**0.0210**	0.0070
*age5-10*	**0.7000**	0.0302	**0.7535**	0.0484	**0.7153**	0.0365	**0.6117**	0.0504
*age10-18*	**0.6456**	0.0277	**0.7038**	0.0437	**0.6323**	0.0323	**0.6107**	0.0445
*age18-60*	**0.2396**	0.0116	**0.2697**	0.0223	**0.2365**	0.0150	**0.2151**	0.0168
*age60+*	**0.1850**	0.0246	**0.3169**	0.0581	**0.1717**	0.0331	**0.0792**	0.0282

Table 3 shows that the highest incidence rates were observed amongst immigrant tribes, with the only indigenous tribe (the Kunda) exhibiting one of the lowest infection rate across each time period, despite making up over 70% of the population.

**Table 3:** Approximate incidence rates by season for different ethnicities.

	Total (3 years)		Hot, Dry Season		Hot, Wet Season		Cool, Dry Season	
Ethnicity	*Average*	*SE*	*Average*	*SE*	*Average*	*SE*	*Average*	*SE*
*Chewa*	**0.7678**	0.0391	**0.8605**	0.0597	**0.7503**	0.0474	**0.7051**	0.0513
*Kunda*	**0.2451**	0.0106	**0.2733**	0.0176	**0.2410**	0.0125	**0.2245**	0.0151
*Ngoni*	**2.3535**	0.1939	**2.4461**	0.3039	**2.5108**	0.2432	**1.8931**	0.2944
*Nsenga*	**0.0688**	0.0117	**0.0669**	0.0214	**0.0632**	0.0159	**0.0818**	0.0234
*Bemba*	**0.4128**	0.0719	**0.5654**	0.1539	**0.4481**	0.1097	**0.1888**	0.1147
*lenje*	**0.2424**	0.1194	**0.2424**	0.2424	**0.2424**	0.1706	**0.2424**	0.2424
*No Data*	**0.0000**	0.0000	**0.0000**	0.0000	**0.0000**	0.0000	**0.0000**	0.0000

Infection rates observed by gender and cattle ownership were comparable across time periods, with males and cattle owning households exhibiting marginally higher infections rates in comparison to females and households without cattle (Table 4).

**Table 4:** Approximate incidence rates by season for different sexes and cattle ownership.

	Total (3 years)		Hot, Dry Season		Hot, Wet Season		Cool, Dry Season	
Gender	*Average*	*SE*	*Average*	*SE*	*Average*	*SE*	*Average*	*SE*
*Male*	**0.3623**	0.0146	**0.3877**	0.0222	**0.3627**	0.0172	**0.3351**	0.0240
*Female*	**0.3486**	0.0148	**0.4012**	0.0243	**0.3453**	0.0167	**0.3015**	0.0202
**Cattle House**	** **		** **		** **		** **	
*TRUE*	**0.3979**	0.0250	**0.4160**	0.0353	**0.4150**	0.0306	**0.3443**	0.0365
*FALSE*	**0.3464**	0.0133	**0.3901**	0.0205	**0.3410**	0.0143	**0.3124**	0.0189

**Fig 14:**
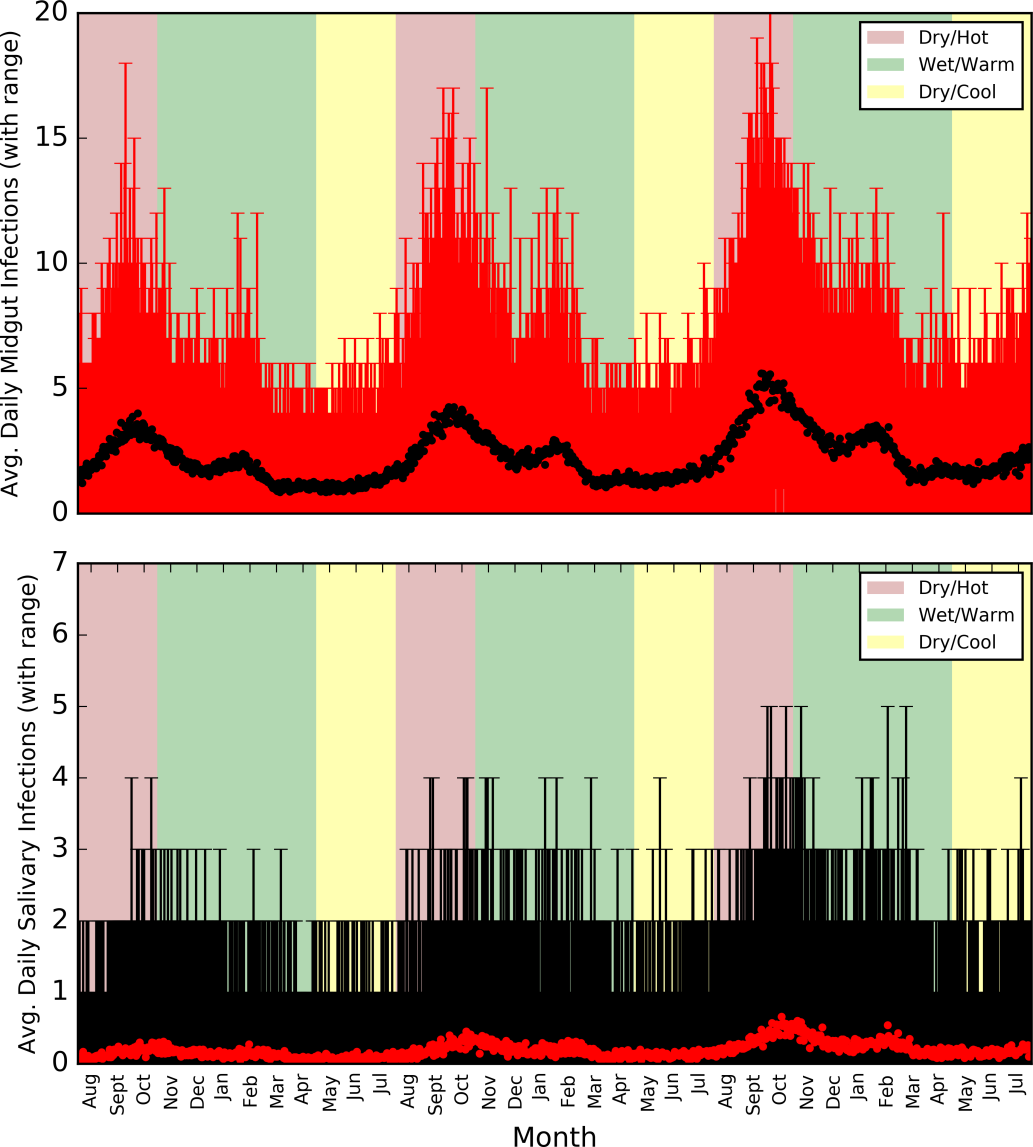
Average and range of tsetse midgut and salivary gland infections as the three year simulation progresses, overlain on a colour-coded background representing the seasons.

Infections acquired and matured within the tsetse population fluctuated as the three year simulation progressed, with a small year-on-year increase in average infections both in the midgut and salivary gland (Fig 14). On average, the peak time of salivary gland infection development was at the beginning of the rainy season, which reflects the period of highest tsetse densities plus a time-lag for development of mature infections in the fly.

## Discussion

### Tsetse population

The first plausible individual-based model representation of a real world tsetse population was created allowing a simulation of the system over multiple years. The model was specified using temperature-dependent parameters derived from the literature, detailed human and animal information from acquired datasets, and expert opinion, and an estimate of the initial tsetse population size and distribution. For example, the pupal population which was completely emergent from the model (as no initial pupae data were inputted) corresponded with literature findings that pupae are comparatively difficult to find in the rainy season, and that the pupal population will be greater than that of the developed flies [38], unsurprising considering that the parameters suggest that pupae are ‘safer’ than teneral flies, pupal duration is at least 3 weeks, and a constant flow of developing pupae is required to replace teneral files which are dying or maturing. In addition, the ratio of female-to-male tsetse fluctuated around 2:1, a change from the simpler, non-seasonal model [27], but more in line with estimates in the literature [67], possibly as a result of running the simulation for longer, and with the addition of climate-driven parameters. The shape of the mature population was comparable to samples of tsetse collected in the region of the South Luangwa National Park (Regional Tsetse and Trypanosomiasis Control Programme (RTTCP) data reported in [22]), Eastern Province, Zambia [68], *G*. *pallidipes* in neighbouring Zimbabwe [61], and similar, yet less detailed, ABM studies [31].

The peak adult population of around 6500 flies suggests that the relatively crude technique used to extrapolate sample data from tsetse surveys for initial model construction (see [27] for more detail) produced a reasonable estimate with 5250 flies. Furthermore, the small teneral population observed is perhaps not a surprise, given that the teneral stage is a brief transition with a gradual input of developing pupae, and high mortality rates coupled with maturation to adult fly on first feed as outputs.

The decrease in pupal population during the rainy season, combined with a consistently small teneral population highlights how one or two years with a very hot and wet rainy season could have serious consequences for a tsetse population, with a reduction in pupal development during periods of high mortality, and high temperatures killing more teneral tsetse reducing the birth rate over subsequent months. Similarly, such a relationship could occur over the coming years in response to climate change, with IPCC reports suggesting that more extreme rainfall events could occur, along with a rise in temperature over the next 50 years (e.g. [57,58]). As a result, it is not surprising that some studies have suggested that certain tsetse fly populations could face extinction within the next 50 years [69]. Future studies will consider using the present model as the basis to test future climate change scenarios and examine the response in the tsetse population to such perturbations.

### rHAT transmission

The model suggested similar incidence rates for rHAT infection in humans and cattle, which is likely to be a response to both the fact that the majority of the cattle were in households at the south of the transect, away from the tsetse zone (only approximately 550 of 2925 cattle were within close proximity of the tsetse zone) [27], and that humans were modelled to be much more active than cattle in the simulation, travelling more frequently away from the home. The latter point is corroborated by similar observations of human incidence rate in both cattle owning and non-cattle owning households, particularly as no human infections occurred while tending to cattle in the field or by the river. As with observations in the previous study, collecting water and school attendance provided the highest proportion of infections by some margin, and is likely to be in response to the high frequency of both trips within the simulation and, for schools, the longer distances travelled to a sparse resource, and the time of day of the trips coinciding with tsetse activity. In support of these simulated observations, a recent study of rHAT infections in Zambia found that almost half of the observed female infections were found in school-age children [70]. The data for males suggested fewer infections in children. This perhaps reflects that school attendance in the model is overestimated for the male population, and, in reality, young men may be needed to work to provide for the family at a younger age. The high incidence rates observed in immigrant tribes gives weight to the suggestion that as populations move down the plateau and into the valley, people are increasingly occupying marginal land, and increasing their exposure to the tsetse fly. As a result, future studies using the model will look to investigate how influxes of people into the region and the associated development affects the tsetse fly population in terms of habitat availability, but also how infection patterns respond to the perturbation of the system.

Six cattle infections were used to seed the model at the beginning of the simulation (along with five goats, one dog and two pigs), to reflect the estimated prevalence of *T*. *b*. *rhodesiense* in the sample of animals from the study transect. The model was also implemented with 10 humans infected at the simulation start, to take into account information from medical teams in the region, who suggested that there had been two reported cases in the past year, and the known high levels of under-reporting and under-detection in the area, and further afield (e.g. [22]). For example, one study suggested that levels of under-detection of rHAT could be as high as 12 cases for every one identified [71]. Furthermore, the recent study in Zambia found that, when a period of more active surveillance was adopted, the number of diagnoses increased dramatically, suggesting high levels of under-detection in the region. In addition, the investigation found that no action was taken by approximately one quarter of people showing symptoms of rHAT infection prior to diagnosis in the study, and less than half sought medical care from a health facility on first sign of symptoms [70]. As a result, given that there is no under-detection in a simulation, two cattle and 11 human infections on average per year appears plausible, especially when considering that there is currently no removal of infection from the simulation (and no reduction in activity when infected), creating a gradually increasing reservoir of infection, and an increase in tsetse infections (Fig 14).

Despite extensive effort to incorporate seasonality accurately into the simulated system, there are some omissions which were largely unavoidable here, but which should be noted. Firstly, in reality, the spatial distribution of tsetse will change through the seasons, with tsetse concentrated in the dense woodland vegetation in the hot dry season, and more widely dispersed in the wet and cool seasons since tsetse use microhabitats to evade extremes in temperature [60,72]. Using an interpolated temperature gradient across a study area through time may allow this behaviour to be simulated, although there would be limitations as temperatures would not reflect sheltered areas utilised by tsetse. As a result, such an implementation should be used in conjunction with a variable land classification, highlighting changes in vegetation with seasons. In addition, no data were available on how human movements vary seasonally in this region at the temporal resolution being modelled, and therefore, the daily routines used are consistent through the year. Finally, it is understood that maturation rate and transmissibility of trypanosomes in tsetse varies with temperature [60], with early work in Zambia suggesting that higher trypanosome infection rates occurred in *G*. *morsitans* in the hot season than in the cold season [54]. However, very little research has been carried out on this subject and, within this study, transmission rates should be low enough for this to have little impact.

### Bigger picture

For the first time in the field of rHAT transmission research, data produced and relationships identified in different studies, focusing on different aspects of the tsetse life-cycle and tsetse-climate interactions, have been incorporated into a single detailed ABM, creating a plausible, stable model, which can ultimately produce a reasonable estimate for transmission rates. While providing an element of validation to these individual entomological studies, through the production of tsetse population curves which closely follow those produced from data collected in the nearby South Luangwa National Park, the model represents a step towards a greater understanding of disease transmission for rHAT in this case, while also being adaptable to gHAT foci in the future. As with all models, the ABM is not without its limitations, for example, variability in tsetse feeding behaviour and preference has been incorporated, but at a basic level. However, through working towards an accurate model representation of the disease landscape one can expect to achieve a greater understanding of the rHAT transmission system, which in turn can help the devising of spatially and temporally targeted mitigation strategies in the future, to help those in need with sustainable solutions, and are more appropriate for spatially marginal communities susceptible to neglected tropical diseases.

### Conclusion

The dynamics of a tsetse population are difficult to model due to difficulties in acquiring data, and the complexity of the system, but are important to understand due to their importance in rHAT transmission. Gaining a greater understanding of tsetse population dynamics may lead to greater understanding of rHAT transmission and aid future mitigation strategies. This paper presented the first seasonally-varying rHAT transmission model, defined at a fine resolution and modelling directly individual flies, with the full tsetse life cycle as a sub-component. By incorporating numerous parameters estimated from the literature, from data and from expert opinion into such a detailed model, a range of outputs were created which can be used by scientists to analyse and evaluate our current understanding of tsetse fly dynamics and the rHAT disease transmission system, and by decision-makers to investigate alternative mitigation strategies. In its current state, including seasonally varying effects, the model lends itself to modelling future scenarios, including insecticide application and other vector control strategies, the incorporation of a changing climate, the effects of landcover change and human development adjacent to, and within, the biodiverse tsetse habitat.
